# Dynamics of beneficial epidemics

**DOI:** 10.1038/s41598-019-50039-w

**Published:** 2019-10-22

**Authors:** Andrew Berdahl, Christa Brelsford, Caterina De Bacco, Marion Dumas, Vanessa Ferdinand, Joshua A. Grochow, Laurent Hébert-Dufresne, Yoav Kallus, Christopher P. Kempes, Artemy Kolchinsky, Daniel B. Larremore, Eric Libby, Eleanor A. Power, Caitlin A. Stern, Brendan D. Tracey

**Affiliations:** 1 Santa Fe Institute, Santa Fe, NM 87501 USA; 2 School of Aquatic and Fishery Sciences, University of Washington, Seattle, WA 98105 USA; 3 Arizona State University, Tempe, AZ 85281 USA; 4 Vermont Complex Systems Center, University of Vermont, Burlington, VT 05401 USA; 5 Massachusetts Institute of Technology, Cambridge, MA 02139 USA; 6 Department of Mathematics and Mathematical Statistics, Umeå University, Umeå, 901 87 Sweden; 7 Oak Ridge National Laboratory, Oak Ridge, TN, 37831 USA; 8 Max Planck Institute for Intelligent Systems, Tübingen, Germany; 9 London School of Economics and Political Science, London, United Kingdom; 10 Melbourne School of Psychological Sciences, Melbourne, Australia; 11 Departments of Computer Science and Mathematics, University of Colorado Boulder, Boulder, CO, 80309 USA; 12 Department of Computer Science and BioFrontiers Institute, University of Colorado Boulder, Boulder, CO, 80309 USA; 13 Department of Methodology, London School of Economics and Political Science, London, United Kingdom

**Keywords:** Theoretical ecology, Evolutionary theory

## Abstract

Pathogens can spread epidemically through populations. Beneficial contagions, such as viruses that enhance host survival or technological innovations that improve quality of life, also have the potential to spread epidemically. How do the dynamics of beneficial biological and social epidemics differ from those of detrimental epidemics? We investigate this question using a breadth-first modeling approach involving three distinct theoretical models. First, in the context of population genetics, we show that a horizontally-transmissible element that increases fitness, such as viral DNA, spreads superexponentially through a population, more quickly than a beneficial mutation. Second, in the context of behavioral epidemiology, we show that infections that cause increased connectivity lead to superexponential fixation in the population. Third, in the context of dynamic social networks, we find that preferences for increased global infection accelerate spread and produce superexponential fixation, but preferences for local assortativity halt epidemics by disconnecting the infected from the susceptible. We conclude that the dynamics of beneficial biological and social epidemics are characterized by the rapid spread of beneficial elements, which is facilitated in biological systems by horizontal transmission and in social systems by active spreading behavior of infected individuals.

## Introduction

Epidemiology has traditionally focused on the spread of harmful contagions, including human viruses such as influenza or dengue fever^1–3^, chytrid fungus in frogs^4^, and bacterial wilt in beans^5^. The serious consequences of detrimental epidemics drive their study, but *beneficial* elements could also spread contagiously, and comparatively little is known about their dynamics^6^. While the social sciences have studied the spread of beneficial behaviors, such as good health practices^7^ or adoption of agricultural technology^8^, there is relatively little mathematical work studying these observed phenomena and connecting them with epidemiological processes.

Beneficial epidemics involving the spread of viruses, plasmids, genes, and microbes, have been identified in biology. For example, beneficial viruses that enhance—or are essential for—survival of a host^9,10^ have been found in both unicellular and multicellular organisms^11^. In addition, many beneficial genetic elements have been identified that spread horizontally among unicellular organisms^12–18^. The dynamics of such biological beneficial epidemics have been investigated in a small number of specific, unstructured populations^12,19,20^, but many open questions remain^6,12,15^.

In the behavioral sciences, social epidemics of behaviors, ideas, and technologies have long been studied (e.g. *Diffusion of innovations* by Rogers^21^, first published in 1962). Examples include the adoption among humans of new agricultural technologies^8^, linguistic variants^22^, and social movements^23,24^, and extend to the acquisition of new feeding techniques among blue tits^25^ and humpback whales^26^. In particular, studies have considered the influence of static network topology on spreading dynamics^8,23,24,27–30^, but the simultaneous dynamics of networks and spreading remain largely unexplored.

**Figure 1:**
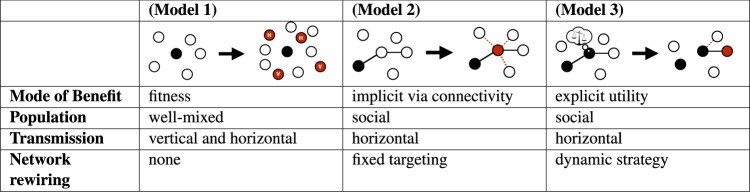
Comparison of bene models. In the three schematics the black arrow represents one increment of time, the black circles are infected individuals, the red circles are newly infected individuals, and the open circles are susceptible individuals. In Model 1 H represents infection from horizontal transmission and V from vertical transmission. In Model 2 dashed red lines indicated new social connections and solid black lines indicated existing connections. The same holds for Model 3, with the addition of strategic rewiring, which includes both adding new links and severing certain existing ones.

We investigate epidemics of beneficial elements, which we call *benes*, in three contexts (Fig. 1). Our goal is to provide a breadth-first modeling exercise for beneficial epidemics in situations of differing dynamics and transmission structure. In many cases our analysis simply involves considering models in atypical parameter regimes. In each context, the bene always provides some benefit to individual hosts, yet the manifestation of the benefit is different and relevant to the specific scenario. In the first context we consider an evolutionary/population genetics model where the benefit is simply increased reproductive fitness. In the context of behavioral epidemiology, a relevant benefit must be manifested within the same generation and affect social behavior. We thus analyze a bene that causes the formation of new network links that preferentially target uninfected nodes. In the context of dynamic social networks with individual agent preferences, the concept of benefit must incorporate the opinions of individuals about what is beneficial to them. In this context, we investigate a family of benes that incite individuals to form new social ties and break existing ones. By studying different types of benefits across these contexts, we can identify features common to the dynamics of beneficial epidemics in general. Ultimately, we find that the spreading dynamics of benes is qualitatively different than in traditional epidemics.

## Model 1: Epidemics with fitness benefits

In biological systems, beneficial mutations increase the reproductive fitness of an organism, increasing the number of offspring the host leaves in subsequent generations^31,32^. Here we consider a beneficial sequence of genetic material, like a virus or plasmid, that also spreads horizontally through the population, and contrast its spread with that of a beneficial element that is only transmitted vertically^33^.

We consider two types of individuals: those infected by the bene, *I*, and those uninfected by the bene and therefore susceptible, *S*. The bene is assumed to increase the reproductive rate of infected individuals by a factor $(1+s)$ where $s > 0$. On its own, a reproductive rate advantage would cause the infected population to eventually outnumber the susceptible population, but in this model, the bene can also spread horizontally between individuals, and so *S* entities are also converted into *I* entities within the same generation (see Supplemental Text for consideration of a fixed population model). We assume that the bene is transmitted across generations with probability $p\in [0,1]$. The growth and infection processes are captured by the time evolution of the *S* and *I* population sizes:

1 $$\dot{S}=S-\beta SI+(1+s)(1-p)I,\,\dot{I}=(1+s)pI+\beta SI,$$

where *β* is the infection rate ($\beta \ge 0$). This basic model is similar to S-I models and work done previously (e.g.^34^).

In the absence of horizontal transmission ($\beta =0$) and assuming vertical transmission is perfect ($p=1$), (1) can be solved analytically: $S(t)=S(0){e}^{t}$ and $I(t)=I(0){e}^{(1+s)t}$. The proportion of the population infected at time *t* is therefore $1/(1+{Z}_{0}{e}^{-st})$, where ${Z}_{0}=S(0)/I(0)$, and as expected, the fraction of uninfected individuals shrinks exponentially. However, with horizontal transmission, $\beta  > 0$, the population is taken over by infected individuals much faster than without horizontal transmission. Consider a slight variant of (1), where susceptible individuals that become infected through horizontal transfer are removed from the susceptible population, but do not add to the infected population, i.e. $\dot{S}$ is the same but $\dot{I}=(1+s)pI$. Under this assumption, the number of infected individuals is the same as without horizontal transfer ($\beta =0$) and the number of susceptible individuals becomes

2 $$S(t)=S(0){e}^{t-\frac{\beta }{1+s}{e}^{(1+s)t}+\frac{\beta }{1+s}}.$$

The proportion of the population uninfected at time *t* is therefore

3 $$Z(t)={[1+{Z}_{0}^{-1}{e}^{\frac{\beta }{1+s}({e}^{(1+s)t}-1)}{e}^{st}]}^{-1},$$

exhibiting a superexponential decay. This is an upper bound on *Z*(*t*) for the full model (Eq. (1)) which means that the susceptible population decreases at least superexponentially.

If vertical transmission is imperfect ($p < 1$) then the infected *I* population continually generates susceptible individuals. The *S* population approaches a steady state of ${S}^{\ast }=(1-p)(1+s)/\beta$ as $t\to \infty$. If we assume that the infected population is initially small, then the system has three dynamical regimes (see Supplemental Fig. S1 for example). First, when $\beta SI\ll S,I$, the population is so dilute that horizontal transmission events are rare, and both populations grow exponentially such that $S\propto {e}^{t}$ and $I\propto {e}^{(1+s)pt}$. As *βSI* increases there is a sharp transition phase where susceptible individuals are rapidly infected. This leads to the final phase where the *S* population approaches the steady state and the *I* population grows exponentially at a rate $I\propto {e}^{(1+s)t}$ that is independent of the vertical transmission probability. Of the three dynamical regimes, the transition phase is the only one with potential for super exponential dynamics since the *I* population is moving from one exponential growth rate to another, larger one.

## Model 2: Epidemics with connectivity benefits

In the previous section, we considered benes whose beneficial effect occurs across generations in a well-mixed population, but in a heterogeneous population, the spread of a bene can be affected in the same generation by a change in the social behavior of infected individuals. Many modes of benefit manifest indirectly in an increased social connectivity of infected individuals: increased energy allowing more social connections, increased social desirability attracting new contacts, or conscious desire to spread the bene. Initially, we do not explicitly model the underlying benefit, but only consider its indirect effect on the network of social contacts in a population. We obtain a preliminary view of the spreading dynamics we expect for such benes. A more explicit consideration of how a contagion’s benefit might induce a change in social behavior is the basis of the model analyzed in the next section.

Harmful contagions can also induce behavioral effects that increase its spread, and the interplay between an infectious disease and changes in the underlying network structure has been studied at great length^35–42^. However, because the contagion is detrimental to the host, there is usually a tension between behavior of infected individuals, affected by the contagion to try to increase its spread, and that of the uninfected population, attempting to limit it. The spread of a purely beneficial contagion would not involve this tension and we expect different spreading dynamics.

We consider an “SIS” model where nodes can be either infected or susceptible, and may transition from either state to the other. Susceptible nodes are infected at a transmission rate *β* by each of their infected neighbors and infected nodes recover at rate *r* to become susceptible. In typical epidemiological applications, we could set $r=1$ without loss of generality and we would expect $0 < \beta  < 1$. On top of this classic model, we suppose that the consequence of the bene is to generate Δ new links upon infection and remove the same amount upon recovery. We also suppose the targets of new links to be chosen either randomly from all nodes in the network or preferentially chosen from susceptible nodes (disassortative). This preference is modeled with the parameter *α* that denotes the assortative bias. When $\alpha =0$, susceptible nodes are always selected as the target for new links by infected nodes. When $\alpha =1$, there is no bias, and targets are chosen uniformly from the population. In between, susceptible nodes are preferentially targeted, but links of both types can be created.

Let *S* and *I* denote the fraction of nodes currently susceptible and infected, respectively, such that $S+I=1$. Let [*SI*] be the number of edges between *S* and *I* nodes normalized by the total population size, and so on for [*SS*] and [*II*]. Following  existing methods^35,36^ for networks with Poissonian degree distribution of mean *k*_0_, we can write the differential equations governing this process as follows.

4 $$\begin{array}{rcl}{\dot{I}} & {=} & {-\,\dot{S}=\beta [SI]-rI}\\ {[\dot{S}S] }& {=} & {-\,2\beta \,([SS][SI]/S)+r[SI]\frac{{k}_{I}-\Delta }{{k}_{I}}}\\ {[\dot{S}I]} & {=} & {2\beta ([SS][SI]/S)-\beta ({[SI]}^{2}/S)-\beta [SI]-r[SI]}\\  &  & {+\beta [SI]\Delta \frac{S}{S+I\alpha }+2\,r\,[II]\frac{{k}_{I}-\Delta }{{k}_{I}}}\\ {[\dot{I}I]} & {=} & {\beta ({[SI]}^{2}/S)+\beta [SI]+\beta \,[SI]\,\Delta \frac{I\alpha }{S+I\alpha }-2r[II],}\end{array}$$

The average degree of an infected node is ${k}_{I}=(2[II]+[SI])/I$. The critical transmission rate for the bene to spread epidemically is then ${\beta }_{c}=r/(\tau +\sqrt{{k}_{0}+{\tau }^{2}})$, where $\tau =\frac{1}{2}({k}_{0}+\Delta -1)$. When $\beta  < {\beta }_{c}$, any small infection dies out and the only stable state is when the entire population is susceptible ($S=1$). When $\beta  > {\beta }_{c}$ the $S=1$ equilibrium becomes unstable and an arbitrarily small infected population will grow to an extensive size.

In a static Poissonian network, the epidemic threshold *β*_*c*_ is simply $r/{k}_{0}$. Notice that while we recover this result in the limit $\Delta \to 0$, our critical transmission rate is not simply that of a Poissonian network with average degree ${k}_{0}+\Delta$. On the one hand, the degree distribution of infected node is of smaller variance than a Poisson distribution, which *raises* the epidemic threshold. On the other hand, there is a feedback between the expected epidemic size and the average degree of the network which *lowers* the epidemic threshold. Our steady state analysis is illustrated in Fig. S6 as a function of model parameters.

We saw in the biological model that the steady state proportion of uninfected individuals can decrease toward zero superexponentially due to a combination of a fitness disadvantage and horizontal transmission. Using the present model, we find that such a superexponential decrease can also occur due to a combination of horizontal transmission and targeted link generation.

If new links are perfectly targeted at susceptible individuals ($\alpha =0$), then as long as more than one link on average is generated per infection ($\Delta \ge 1$), the susceptible population shrinks double-exponentially, that is, $dS(t)/dt=-\,x(t)S(t)$, where $dx(t)/dt=\beta (\Delta -1)x(t)$. On the other hand, if $\Delta  < 1$, then even if new links are perfectly targeted, the rate at which *S* decreases itself decreases exponentially.

Suppose that infected individuals imperfectly target susceptible individuals. Then, as *I* becomes much larger than *S*, even a small $\alpha  > 0$ causes most new links to be made toward already infected individuals. Effectively, this is equivalent to $\Delta \to 0$. The rate at which *S* decreases will decrease exponentially, as in a standard epidemic process. We expect two phases in the final spreading dynamics: at first, when $\alpha \ll S$, the behavior will be as if $\alpha =0$, with double-exponential decay of the susceptible population size (assuming $\Delta  > 1$). However, eventually *S* becomes smaller than *α*, and the system acts as if no extra *SI* links are generated.

**Figure 2:**
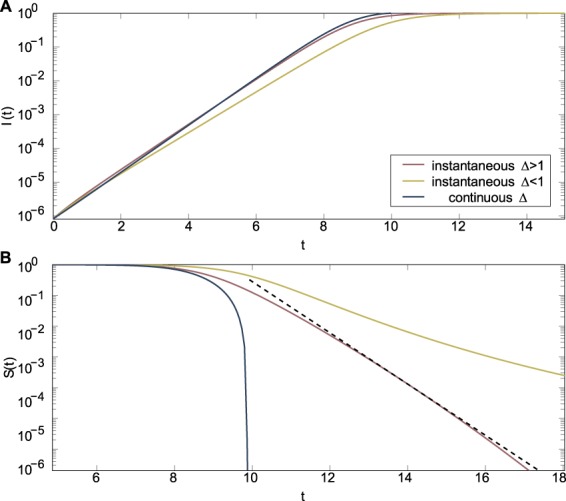
Perfect targeting $(\alpha =0)$ initial and final dynamic regime. (**A**) The fixation dynamics are shown for both the instantaneous and continuous link generation models. In the continuous model the convergence to complete fixation is in finite time. For the instantaneous model, complete fixation is approached faster than exponentially (when $\Delta  > 1$, dotted straight line for comparison) or is not approached even for very long times (when $\Delta  < 1$). (**B**) The initial dynamics is given for the same cases. In all these plots the recovery rate is $r=0$ and the transmission rate $\beta =1/2$, the initial degree ${k}_{0}=3$.

In the Supplementary Information, we analyze a similar model where the extra connectivity accrues throughout the time an individual is infected. We then find that the infection always reaches fixation in finite time. Analytic results are summarized in Table 1 and illustrated in Fig. 2.

**Table 1:** Dynamics of benes with connectivity benefits.

	instantaneous Δ		continuous Δ	
	*α* = 0	*α* > 0	*α* = 0	*α* > 0
epidemic threshold	$\frac{{\beta }_{c}}{r}=\frac{1}{\tau +\sqrt{{k}_{0}+{\tau }^{2}}}$		—	
	$$\tau =\frac{1}{2}({k}_{0}+\Delta -1)$$			
early time	exp. growth; const. rate		exp. growth; variable rate	
fixation	${e}^{-{e}^{t}}$	${e}^{-{e}^{-t}}$	${({t}^{\ast }-t)}^{2}$	*e* ^− *t*^

The behaviors observed in our epidemiological models differ drastically from classic spreading dynamics. In short, the addition of the connectivity benefit affects how the average degree impacts the epidemic threshold, affects the exponential rate of early time spread, and affects the nature of the fixation dynamics which are sensitive to both how these new links are created and to whom. The impact of the benefit on the epidemic threshold and fixation dynamics are the most important because they illustrate how this model of benes on contact networks is not merely a different region of parameter space for classic models of epidemics on networks.

## Model 3: Epidemics with utility benefits

The previous section considered how a bene spreads when its implicit benefit manifests in increased connections. Here, we consider the case when a bene has explicit consequences for an individual’s utility. We call an infectious trait a bene if becoming infected leads to an increase in utility. We consider how the utility conferred by the infection leads individuals to rewire strategically so as to influence infection dynamics and thereby increase their future expected utility. For example, if infected individuals can increase utility by growing the size of the infected population, they can ‘proselytize’ and spread the trait by seeking out new social connections to the susceptibles; on the other hand, if the infected gain utility from only being connected to other infected, the opposite rewiring dynamic can take hold.

We consider an epidemiological model in which both infected and susceptible individuals rewire their connections based on a utility function. The utility function reflects preferences for local conformity versus global spreading of the infection. Infected and susceptible individuals’ utility functions are indicated by ${U}_{I}({I}_{n},{S}_{n},{I}_{g})$ and ${U}_{S}({I}_{n},{S}_{n},{I}_{g})$ respectively, where *I*_*n*_ is the number of infected neighbors, *S*_*n*_ is the number of susceptible neighbors, and *I*_*g*_ is the total number of infected individuals in the global population.

Importantly, individuals rewire based on their predictions of how their future expected utility will change due to epidemic spreading dynamics. In making predictions, individuals only make use of knowledge of their direct connections (and not, for example, connections between their neighbors). We use *P*_*I*_ and *P*_*S*_ for the predicted expected utility of infected and susceptible individuals. As before, transmission is assumed to be a simple contact process with rate *β*.

We show that different preferences for local and global infections lead to different dynamical regimes. Here we assume that the utility functions are linear: ${U}_{I}({I}_{n},{S}_{n},{I}_{g})={a}_{I}{I}_{n}+{b}_{I}{S}_{n}+{c}_{I}{I}_{g}$ and ${U}_{S}({I}_{n},{S}_{n},{I}_{g})={a}_{S}{I}_{n}+{b}_{S}{S}_{n}+$ ${c}_{S}{I}_{g}$, where *a*_*I*_ and *a*_*S*_ are parameters specifying the utility of one additional infected neighbor, *b*_*I*_ and *b*_*S*_ specify the utility of one additional susceptible neighbor, and *c*_*I*_ and *c*_*S*_ specify the utility of increasing the number of infected individuals in the global population by one. We also define ${d}_{I}={a}_{I}-{b}_{I}$ and ${d}_{S}={a}_{S}-{b}_{S}$, the utilities of swapping a susceptible neighbor for an infected one.

Infected individuals’ predictions account for the probability that they will infect some number $X=0,\ldots ,{S}_{n}$ of their susceptible neighbors. Assuming a well-mixed population of neighbors, *X* is distributed as a binomial, $X\sim B({S}_{n},\beta )$, giving

$$\begin{array}{rcl}{P}_{I}({I}_{n},{S}_{n},{I}_{g}) & = & {{\mathbb{E}}}_{X}[{U}_{I}({I}_{n}+X,{S}_{n}-X,{I}_{g}+X)]\\  & = & {U}_{I}({I}_{n},{S}_{n},{I}_{g})+({d}_{I}+{c}_{I})\beta {S}_{n}.\end{array}$$

Susceptible individuals account for the probability that they become infected by at least one of their infected neighbors

$$\begin{array}{ccc}{P}_{S}({I}_{n},{S}_{n},{I}_{g}) & = & (1-{(1-\beta )}^{{I}_{n}}){U}_{I}({I}_{n},{S}_{n},{I}_{g}+1)+\,{(1-\beta )}^{{I}_{n}}{U}_{S}({I}_{n},{S}_{n},{I}_{g})\\  & \approx  & \beta {I}_{n}({U}_{I}({I}_{n},{S}_{n},{I}_{g})+{c}_{I})+\,(1-\beta {I}_{n}){U}_{S}({I}_{n},{S}_{n},{I}_{g}),\end{array}$$

where we use ${(1-\beta )}^{{I}_{n}}\approx 1-\beta {I}_{n}$ assuming $\beta {I}_{n}\ll 1$.

The parameters ${a}_{I},\ldots ,{c}_{S}$ determine individuals’ rewiring behavior. For an infected individual, the change in predicted utility from disassortative rewiring is

$${P}_{I}({I}_{n}-1,{S}_{n}+1,{I}_{g})-{P}_{I}({I}_{n},{S}_{n},{I}_{g})=-\,{d}_{I}+({d}_{I}+{c}_{I})\beta $$

Assortative rewiring is chosen when:

5 $${d}_{I}\ge ({d}_{I}+{c}_{I})\beta $$

while disassortative rewiring is chosen otherwise. For infected individuals, the predicted utility of each rewiring strategy does not depend on the state of the population: either the assortative or the disassortative regime will hold for all infected individuals at all times.

For a susceptible individual, the change in predicted utility from disassortative rewiring is

$${d}_{S}+\beta [({a}_{I}-{a}_{S})\,({I}_{n}-{S}_{n})+2({b}_{I}-{b}_{S}){S}_{n}+({c}_{I}-{c}_{S}){I}_{g}+{c}_{I}]$$

with the negative for assortative rewiring. Thus, for susceptible individuals, the predicted utility of each rewiring strategy depends on the state of the population, and not just the parameters. The assortative rewiring will be chosen when

6 $$({a}_{I}-{a}_{S}+{d}_{I}-{d}_{S}){I}_{n}+({b}_{I}-{b}_{S}){S}_{n}+({c}_{I}-{c}_{S}){I}_{g} < -\,\frac{{d}_{S}}{\beta }-{c}_{I}+({d}_{I}-{d}_{S})$$

and disassortative otherwise.

This framework tells us how individual preferences translate into assortative or disassortative rewiring behavior for infected and susceptible individuals in the course of an epidemic. This allows us to formulate dynamics similar to those presented in the earlier sections (see Supplemental Text C for the general derivation), but now derived from the preferences and predictions of individuals. By combining epidemiological modeling with strategic rewiring, our framework could be used to analyze social movements, the spread of technologies, and strategic rewiring dynamics in detrimental infections (e.g., in which individuals rewire to avoid infection^43,44^).

We now consider three illustrative cases, corresponding to three different sets of parameter values. In the *evangelizers* case, the utility of infected individuals increases when the infection spreads globally, corresponding to ${c}_{I}=1$ (non-specified parameters are 0). In this case, infection causes an increase in utility and is thus a bene. Based on (5) and (6), infected as well as susceptible individuals rewire disassortatively, tempted by the possibility of increasing global spread. In the *cool kids* case, all individuals prefer increasing the number of infected neighbors (the “cool” kids) and decreasing the number of susceptible neighbors (the “uncool” kids), corresponding to ${a}_{I}={a}_{S}=1$ and ${b}_{I}={b}_{S}=-\,1$. In this case, infection confers no direct net utility change. Yet, infected individuals rewire assortatively, while susceptibles rewire disassortatively. Finally, in the *snobs* case, infected individuals prefer to be connected to other infected individuals, while susceptibles are indifferent, corresponding to ${a}_{I}=1$, ${b}_{I}=-\,1$. In this case, whether or not infection causes an increase in utility depends on an individual’s neighborhood. As a result, susceptibles exhibit a complicated behavior: they switch from assortative to disassortative behavior at a particular cutoff number of infected neighbors ${I}_{n} > \frac{{S}_{n}+2}{3}$. As long as susceptible neighbors are numerous, susceptible individuals are assortative to avoid the risk of an infection, which would put them at odds with their neighborhood. Once infected neighbors become sufficiently numerous, the susceptible become disassortative to have the chance to become infected and conform.

These three cases are motivated by previously studied social processes. The evangelizers case can be seen as a model of explicit recruitment in a social movement^24,45^. The cool kids case reflects the transmission of an idea through a group, where infected individuals’ assortativity results in the formation of cliques (e.g., the anticonformity copying modeled in^46^). The snobs case is related to models of segregation^47,48^ where potentially asymmetric and conflicting preferences for assortativity exist.

We simulate the dynamics of this model with ODEs (Supplemental Text C) similar to the ones in the previous sections. In addition to contact spreading dynamics, assortative and disassortative rewiring is performed according to the rules described above. We assume a well-mixed compartmental model with *N* individuals and *E* edges, in which we track the proportion of infected individuals *I*, and the proportion of [*II*], [*SI*], and [*SS*] links. Neighborhoods (i.e. values of *I*_*n*_ and *S*_*n*_) are assumed to be drawn with replacement from these compartments.

**Figure 3:**
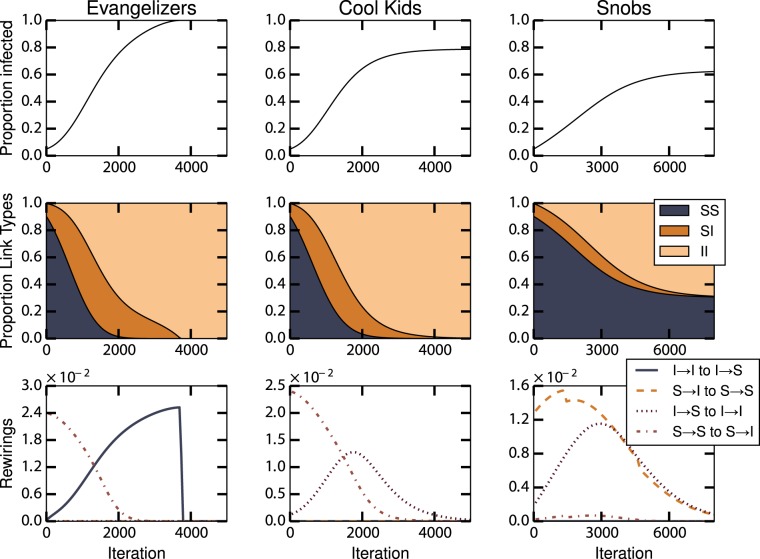
Dynamical regimes for three different models of epidemics with utility benefits. The first row shows the number of infected individuals over time. The second row shows proportion of [*II*], [*SI*] and [*SS*] over time. The third row shows the rate of different rewirings, where for example, ‘I → I to I → S’ indicates the rewiring by an infected individual from an infected to a susceptible. Here $N=1000$, $E=2000$, ${I}_{init}=0.05$, and $\beta =5\times {10}^{-4}$.

Figure 3 shows the dynamics for each case. The evangelizers case exhibits the same superexponential fixation dynamics as in the epidemic model with connectivity benefits (see Supplemental Text C). The disassortative behavior of both infected and susceptible individuals speeds up the epidemic and drives the fixation dynamics. In the cool kids case, the epidemic is incomplete. The susceptible rush to rewire to the infected, as shown by initially high rates of ‘S → S to S → I’ rewiring, while the infected break ties with the susceptible, as seen by the increase in the rate of ‘I → S to I → I’ rewiring once the number of infected individuals rises. These two behaviors compete, but once the infected are sufficiently numerous the latter dominates and the doors to the infected community close. In the resulting network, all connections are between infected individuals ($[II]=1$), susceptibles are isolated, and the epidemic halts. In the snobs case, the epidemic reaches even fewer individuals than in the previous case. While the infected rewire assortatively, the susceptible have a mix of strategies. The result is that the network divides into two completely disconnected components ($[SI]=0$ and $[II]+[SS]=1$), preventing the epidemic from reaching the whole population. We also implemented this model using an explicit agent-based simulation, which included effects of stochasticity, local network heterogeneity, and correlations of connectivity properties across the network. The results were qualitatively similar to the ODE model results reported here (Supplemental Text C and Fig. S7).

**Figure 4:**
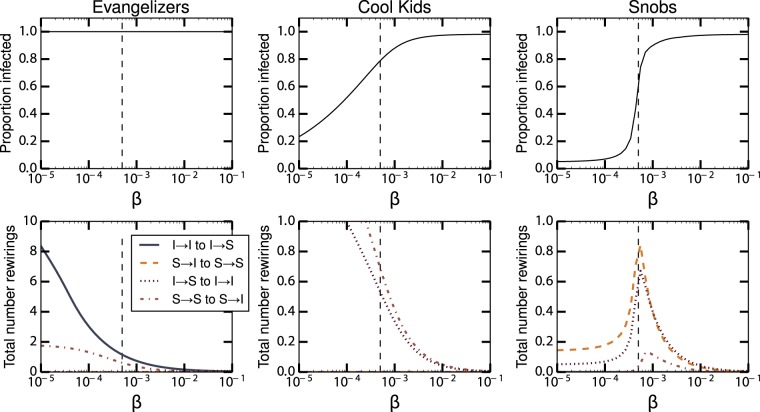
Results of strategic rewiring epidemics with varying transmission rates *β*. The top row shows the proportion of infected for the three cases discussed in the text. The bottom row shows the cumulative rewiring performed (per edge). The value of *β* used for Fig. 3 is shown as a vertical dashed line.

The characteristics of these regimes vary with *β*. Figure 4 shows the final reach of the epidemic and the cumulative rewiring for different values of *β*. For the evangelizers case, the epidemic always spreads to the entire population. For the cool kids case, the reach of the epidemic increases gradually with *β*, since faster spreading increases how many individuals get infected before the susceptible become disconnected. For the snobs case, larger *β* increases the reach of the epidemic, with a rapid transition from minimal spread to full spread once *β* passes a critical threshold. In all these cases, the total amount of rewiring decreases when *β* is sufficiently large, since rewiring stops once the epidemic has swept through the population. Interestingly, the amount of rewirings for the snobs case also decreases with lower *β*, peaking around the critical threshold. This occurs because when transmission is slow, equilibrium is reached very quickly: assortative rewiring by the infected and the susceptible quickly disconnects these two groups before the infection can spread.

This model shows that strategic rewiring affects epidemic dynamics in multiple ways. When infected individuals benefit from increasing the global number of infections, it leads to an accelerating uptake and a much faster global spread than an epidemic without strategic rewiring. If instead the infected individuals tend to assort, the epidemic can be stalled as infected individuals entirely disconnect from susceptible ones.

## Discussion

In this paper, we model different scenarios of beneficial contagions, or benes. We investigate benes in several distinct systems, both biological and social. While the dynamics by which benes spread depend on the particular benefit conferred, we find commonalities across these systems. One interesting outcome is that all scenarios exhibit superexponential fixation in particular regimes, caused by both new regions of parameter space in traditional models (see Model 1) or by new mechanisms outside the scope of traditional models (see Model 2 and 3).

A prime example of superexponential behavior is found in our evolutionary model. Here, the bene confers a fitness advantage to infected individuals, and, in contrast to a standard positive mutation, the bene can also be transmitted across individuals within a generation. One example of such a bene would be antibiotic-resistance cassettes^49^, where bacteria acquire genes from neighboring cells that increase survival when exposed to antibiotics. Our model shows that horizontal transfer of such elements, even when vertical transmission is imperfect, dramatically reduces the time required to fully infect the population. The superexponential decrease in the susceptible population occurs when $\beta  > 0$ and $s > -\,1$. It should be noted that this parameter range corresponds to benes (where $s > 0$) as well as weakly detrimental elements (where $-\,1 < s < 0$). Both spread superexponentially because of the dual modes of transmission, and thus, one needs to be careful in distinguishing benes from detrimental elements.

The importance of horizontal transfer in evolutionary processes led us to consider a bene which increases interactions between individuals in a network. An example is new technologies with network effects, like the file-sharing service Dropbox, that incentivize users to actively recruit new members. We use an “SIS” epidemiological model to analyze the effects of these added network links. We find that added connections change how the epidemic threshold is determined by network density, allowing benes to break out despite lower transmissibility. We also find a much lower fixation time within the population. In fact, if new edges are added only with susceptible individuals, the bene sweeps the entire population in finite time. This result demonstrates that individual behavior is important in determining whether a beneficial epidemic occurs.

In the model of epidemics with utility benefits, individuals’ behavior is based on preferences for the distribution of the infection in the local neighborhood and global population. They strategically rewire based on predictions about how their actions will increase utility. One example is the phenomenon called NIMBY^50^, where individuals have a preference for global adoption of a technology but do not want it in their immediate neighborhood, e.g. wind turbines. As we show in three illustrative cases, variation in the strength of these preferences leads to different dynamical regimes. In one regime, displayed by the *evangelizer* case, infected individuals rewire to susceptible ones, facilitating the bene’s spread. Social movements instilling the desire to convert anybody, and not just acquaintances, will spread quickly. In contrast, when individuals prefer to conform with their neighbors, as in the *cool kids* and *snobs* cases, assortative rewiring results in a disconnected network and a stalled epidemic. Thus, the outcome of an epidemic may reveal the mechanisms underlying its generating dynamics.

It should be noted that our efforts provide a set of testable predictions regarding the dynamics of beneficial epidemics, and could be tested in a variety of contexts. For example, one could test the rate of spreading of a novel beneficial gene in a bacterial lineage as a function of both the degree of horizontal gene transfer and strength of the benefit. In many cases the observation of superexponential dynamics will require high resolution temporal data to accurately categorize a bene and the associated model parameters.

In this paper we considered the dynamics of beneficial epidemics for certain biological and social systems. We investigated contagions that confer specific types of benefits related to fitness or social utility, but many other types of beneficial epidemics are possible. For example, one could combine elements of our three models so that changes in social networks have cross-generational effects. Alternatively, one could consider a contagion that is beneficial to one type of host but harmful to others. These more complex models may exhibit other interesting behaviors that differ from the more traditionally and extensively studied harmful epidemics. By differentiating between the dynamics of various types of epidemics, it may be possible to identify distinct signatures of epidemics and determine the type of contagion as it is spreading in real time.

## Supplementary information

Supplementary Text and Figures
