# Supplementary material for: Dynamics of beneficial epidemics
Source: Sci Rep. 2019 Oct 22;9:15093. doi: 10.1038/s41598-019-50039-w (PMC6805938; doi:10.1038/s41598-019-50039-w)
Supplement: Supplementary file 1 — Supplementary Text and Figures [file 41598_2019_50039_MOESM1_ESM.pdf]

Supplementary Information for "Dynamics of beneficial epidemics"

Andrew Berdah, Christa Brelsford, Caterina De Bacco, Marion Dumas, Vanessa Ferdinand, Joshua A. Grochow, Laurent Hébert-Dufresne, Yoav Kallus, Christopher P. Kempes, Artemy Kolchinsky, Daniel B. Larremore, Eric Libby, Eleanor A. Power, Caitlin A. Stern & Brendan D. Tracey

1 Evolutionary model

Imperfect transmission numerical solution We solve the system of equations with imperfect transmission using parameters $\beta = .05$, $s = .01$, and $p = .75$. Thus, there is a small fitness benefit to the bene and it fails to transmit vertically with a probability of .25. Starting with an initial concentration of .01 for susceptible (red) and .0001 for infected (dashed), we see that there are distinct dynamical regimes (Figure S1). First, both populations grow exponentially, then a rapid decline in the susceptible population, followed by steady growth for the infected and no growth for the susceptible population. The bottom panel shows the growth coefficient, i.e. the slope in log space for both populations. Only in middle regime is there the potential for super exponential dynamics.

Fixed population size In the main paper, we considered a bene in an expanding population. Here, consider the same effect in a model with a finite population of size N. We use a previously published model of horizontal gene transfer [47] which is easily generalized to other mechanisms of horizontal transmission of genetic elements found in multicellular organisms such as crustaceans and insects [48]. This model ((1)) distinguishes the effects of the transmission rate β of the bene and the selective value s of the bene. The number of I entities is n and the number of S entities is $N - n$. The model is based on a Moran process [49] in which a birth/death process occurs in discrete time steps. The probability of having n infected types at time t, $p_n(t)$, depends on the cumulative effects of the birth (λ_n) and death (μ_n) rates (see (1)). The birth rate (λ_n) includes actual births (the first term, which involves s) as well as horizontal gene transfer (the second term, which involves β). There are two stationary states of this model: either i) the entire population is un-infected (S), or ii) the entire population is infected (I). We solve for the stationary distribution of $p_n(t)$ starting with $p_n(0) = \delta_{n,1}$ (i.e all realizations start with a single infected individual ($n = 1$)).

$$
\begin{aligned}
\frac{dp_n}{dt} &= \mu_{n+1}p_{n+1} - (\lambda_n + \mu_n)p_n + \lambda_{n-1}p_{n-1} \\
\lambda_n &= (1+s)n\frac{N-n}{N+1} + \beta\frac{n(N-n)}{N} \\
\mu_n &= (N-n)\frac{n}{N+1}
\end{aligned}
\tag{1}
$$

Figure S2 shows the fixation probability and time to stationarity as a function of β for two

example values of s. As β increases, the probability that the bene fixes increases (left panel). Higher transmission rates also lead to faster time to a stationary solution when compared to those achieved by selection alone (right panel). The decrease in time to reach a stationary solution is more dramatic when selection ($s = 0.1$) is lower. Thus for more modest fitness values of the bene the role of horizontal gene transfer is greater.

In Figs. SS3-S5, we investigate the interplay of horizontal and vertical transmission over different ranges of parameter values and temporal intervals. We also test the validity of our closed-form solution, which was obtained in a special regime but appears to be a good approximation of the true dynamics at high bene fitness.

2 Epidemics with connectivity benefit

Dynamical equations In the dynamical system describing the spread of a bene with connectivity benefits, we use S and I to denote the fraction of nodes susceptible and infected, respectively, at a given time. We use $[SI]$ to denote the number of edges between S and I nodes normalized by the total population size, and so on for $[SS]$ and $[II]$. We also define such variables for triplets, such that, for example, $[ISI]$ is the number of node triplets, such that one of which is susceptible and has edges connecting it to the other two, which are infected.

In the limit of a large population, the change in S, I, $[SS]$, $[SI]$, and $[II]$ over time is given by ordinary differential equations

$$\begin{aligned}
\dot{I} &= -\dot{S} = \beta[SI] - rI \\
[\dot{SS}] &= -\beta\,[SSI] + r[SI]\frac{k_I - \Delta}{k_I} \\
[\dot{SI}] &= \beta[SSI] - 2\beta[ISI] - \beta[SI] - r[SI] + \\
&\quad \beta[SI]\Delta\frac{S}{S + I\alpha} + 2\,r\,[II]\frac{k_I - \Delta}{k_I} \\
[\dot{II}] &= 2\beta[ISI] + \beta[SI] + \beta\,[SI]\,\Delta\frac{I\alpha}{S + I\alpha} - 2r[II],
\end{aligned} \tag{2}$$

where $k_I = (2[II] + [SI])/I$ is the average degree of an infected node.

Moment closure To solve the dynamical equations (2), we need to determine the triplet densities $[ISI]$ and $[SSI]$. We could write down differential equations for their evolution, but those would involve still new terms specifying the density of four-node motifs. Therefore, we use a moment-closure approximation to express these triplets in terms of the previously defined pairs. We do this by assuming that if a node of type X has k incident edges, each of those edges is independently taken to be an $[XY]$ node with probability proportional to $[XY]$ if $Y \neq X$ and $2[XX]$ if $Y = X$.

Therefore, the concentrations of triplets that feature in the differential equations are given by

$$\begin{aligned} [ISI] &= \gamma_S \frac{[SI]^2}{2S} \\ [SSI] &= \gamma_S \frac{2[SS][SI]}{S} \\ \gamma_S &= \frac{\langle k^2 \rangle_S - \langle k \rangle_S}{\langle k \rangle_S^2} \end{aligned} \tag{3}$$

The factor γ_S compensates for the excess degree of an S node, taking into account the fact that the expected number of additional edges a node has conditioned on having at least one edge is not necessarily the same as the unconditional excepted number of edges. For a Poisson degree distribution, they are the same, and that factor is 1. Because, in our graph, edges are continually being created and destroyed, γ_S would also be changing over time, not only because the average degree $\langle k \rangle_S$ would be changing, but also because the continual redistribution of edges would drive the network toward a Poissonian degree distribution. To avoid tracking the changes in the degree distribution, we assume a Poissonian distribution for the susceptible nodes at all times, $\gamma_S = 1$.

Outbreak dynamics Plugging the moment closure (3) into the dynamical equations (2), we obtain a system of four coupled differential equation in four variables, $I(t)$, $[SS](t)$, $[SI](t)$, and $[SS](t)$, and with parameters denoted by k_0, Δ, β, r, and α. We note that the equations satisfy the conservation law

$$\dot{[SI]} + \dot{[SS]} + \dot{[II]} - \Delta \dot{I} = 0, \tag{4}$$

which reflects the fact that the total number of edges in the network is directly related to the number of infected nodes, since each infection event introduces Δ edges, and each recovery removes the same number. Therefore, we can eliminate $[II]$ from the system of equations, replacing it with $[II] = \frac{1}{2}k_0 + \Delta I - [SS] - [SI]$, and be left with three coupled ODEs for three variables.

The state where there are no infected nodes is a fixed point of the system, given by $I = 0$, $[SI] = 0$, and $[SS] = \frac{1}{2}k_0$. To determine whether this fixed point is stable, that is, if a small infection spreads as an epidemic or dies out, we would normally look at the Jacobian of the system of ODEs at the fixed point. However, the Jacobian is singular at this fixed point (note that k_I is not well defined when $I = [SI] = [II] = 0$). In order to properly analyze the stability, we first have to perform a change of variables that resolves the singularity. One change of variable that accomplishes this task is

$$\begin{aligned} z_1 &= \frac{I}{k_0 + 2\Delta I - [SI] - 2[SS]} \\ z_2 &= k_0 + 2\Delta I - [SI] - 2[SS] \\ z_3 &= \frac{\frac{1}{2}k_0 + \Delta I - [SI] - [SS]}{k_0 + 2\Delta I - [SI] - 2[SS]}. \end{aligned} \tag{5}$$

The values of the old variables at the fixed point, $I = [SI] = [SS] - \frac{1}{2}k_0 = 0$, do not determine

the values of z_1 and z_3. So, we solve the equations $\dot{z}_1 = \dot{z}_3 = 0$ to determine the values of z_1 and z_3 at the fixed point.

The Jacobian of the time derivatives, $\dot{z}_1$, $\dot{z}_2$, and $\dot{z}_3$, is generically nonsingular at this fixed point, and its eigenvalues determine the stability of the fixed point. If all eigenvalues are negative, the fixed point is stable. If any eigenvalue is positive, the fixed point is unstable. For any value of the parameters k_0, Δ, r, and α there is a critical value of the transmissibility β_c, such that, if $\beta < \beta_c$, the $I = 0$ fixed point is stable, and if $\beta > \beta_c$, the $I = 0$ fixed point is unstable. We find the critical value to be $\beta_c = r/\left(\tau + \sqrt{k_0 + \tau^2}\right)$, where $\tau = \frac{1}{2}(k_0 + \delta - 1)$. At this value of β, the infection free fixed point is given by $z_1 = \beta_c/(1 + \beta_c)$, $z_2 = 0$, and $z_3 = \beta_c/2(1 + \beta_c)$. The Jacobian can be directly calculated and shown to have two negative eigenvalues and one zero eigenvalue, as expected.

In the case of a contagion without connectivity benefit, i.e. $\Delta = 0$, we recover the classic SIS dynamics and $\beta_c = r/k_0$ [31,32]. However, our result for β_c is not merely the critical transmission rate for a network with Poisson degree distribution of average $k_0 + \Delta$. On the one hand, the degree distribution of infectious individuals is truncated at values below Δ; it is therefore not exactly Poisson for small k_0 and this tends to increase β_c. On the other hand, and more interestingly, the expected degree of susceptible nodes are also growing with the number of infectious nodes. This last detail is crucial: there is a feedback between the expected epidemic size and the connectivity of the network which lowers the epidemic threshold β_c.

Steady-state Convergence When $\beta > \beta_c$, a new stable fixed point emerges with $I > 0$. This represents the stable steady-state value of the infected population, where infections and recoveries occur at the same rate. This value can be obtained by solving the algebraic set of equations given by setting $\dot{I} = [\dot{SI}] = [\dot{SS}] = 0$.

Figure S6 shows the steady-state fraction of infected individuals as a function of β. As β increases, so does the long-term percentage of infected individuals. The figure also depicts the effect of α on the long term percentage. As α decreases, the targeting of infected individuals improves, and thus the fraction of infected individuals increases.

Fixation dynamics under perfect targeting We now consider the dynamics at the conclusion of the epidemic. If we consider the case with no recovery ($r = 0$), then the susceptible population always tends to decrease. The rate of this decrease varies with the value of the assortative bias α and the number of new links generated per infection Δ.

The governing equations in Eq. 4 become simpler in the case where $S \ll 1$ with no recovery. The term $[SS]$ becomes negligibly small as it is second order in the number of S nodes (it requires

two S nodes to be connected to one another). The final term in the $[\dot{SI}]$ equation is

$$\beta[SI]\frac{S}{S+I\alpha} \tag{6}$$

(6) has two regimes with qualitatively different behavior: $\alpha > 0$ and $\alpha = 0$.

(6) becomes 1 for $S \ll I\alpha$. The full system thus becomes

$$\begin{aligned} \dot{S} &= -\beta[SI] \\ [\dot{SI}] &= \beta[SI]\left(-\frac{[SI]}{S} - 1\right) + \beta[SI]\Delta. \end{aligned} \tag{7}$$

The solution of these coupled ODEs can be seen with a variable substitution called x.

$$x = \frac{[SI]}{S} \tag{8}$$

With this definition, we get the following relation:

$$\dot{x} = \frac{[\dot{SI}]}{S} - \frac{[SI]}{S}\frac{\dot{S}}{S} \tag{9}$$

By substituting the evolution equations for $\dot{S}$ and $[\dot{SI}]$, we get an uncoupled equation.

$$\dot{x} = \beta x(-x - 1 + \Delta) - x(-\beta x) = (\Delta - 1)\beta x \tag{10}$$

Using Eqn 7, we get the system of equations:

$$\begin{aligned} \dot{x} &= (\Delta - 1)\beta x \\ \dot{S} &= -\beta x S \end{aligned} \tag{11}$$

In this coupled set of equations, $\log x$ changes at a rate $(\Delta - 1)\beta$, and $\log s$ changes at a rate of βx.

$$\begin{aligned} x(t) &\sim \exp[(\Delta - 1)\beta t] \\ S(t) &= \exp\left(-\beta \int_0^t x(t')dt'\right) \end{aligned} \tag{12}$$

From (12), we see that if $\Delta < 1$, the proportion of S nodes decays at an exponential rate that is decaying exponentially. That is, as S decreases, the rate at which S decreases gets slower and slower. Interestingly, because of the exponentially decreasing rate, even as $t \to \infty$ there are always individuals who are not infected.

On the other hand, if $\Delta > 1$ the rate at which S decreases grows in time. In this regime, S never fully reaches 0, but it tends to 0 more and more quickly as the epidemic spreads. At the critical point, $\Delta = 1$, the rate at which S shrinks is constant.

Fixation dynamics under imperfect targeting If $\alpha > 0$, $\frac{S}{S+I\alpha}$ approaches $\frac{S}{\alpha}$ as S approaches 0. The coupled ODE system reduces to

$$\begin{aligned} \dot{S} &= -\beta[SI] \\ \dot{[SI]} &= \beta[SI]\left(-\frac{[SI]}{S} - 1\right) + \beta[SI]\Delta\frac{S}{\alpha}. \end{aligned} \tag{13}$$

Here, there are two regimes:

$$\begin{aligned} I \gg S \gg \alpha \\ \alpha \gg S \end{aligned} \tag{14}$$

In the first regime where $I \gg S \gg \alpha$, $\frac{S}{S+I\alpha}$ is approximately 1 which means that the behavior is the same as if there is perfect targeting ($\alpha = 0$). However, as the infection proceeds and S gets sufficiently small, the regime switches. As a result $\frac{S}{S+I\alpha}$ is approximately 0, and so the behavior is as if $\Delta = 0$. As S becomes very small, new links to susceptible individuals are added with increasing low frequency. Thus, in the final stages of the epidemic, the additional links added by newly infected individuals only have an impact if they perfectly attach to susceptible individuals.

Continuous link creation We now consider a case where the extra connectivity accrues throughout the time an individual is infected. To keep the analysis simple, we ignore the possibility that an infected node recovers. The system of differential equations describing the system is

$$\begin{aligned} \dot{I} &= -\dot{S} = \beta[SI] \\ \dot{[SS]} &= -\beta\,[SI]\,2\frac{[SS]}{S} \\ \dot{[SI]} &= \beta[SI]\left(2\frac{[SS]}{S} - \frac{[SI]}{S} - 1\right) + I\Delta\frac{S}{S+I\alpha} \\ \dot{[II]} &= \beta[SI]\left(\frac{[SI]}{S} + 1\right) + I\Delta\frac{I\alpha}{S+I\alpha} \end{aligned} \tag{15}$$

Outbreak dynamics In the continuous link-addition model (15), the spread of the epidemic accelerates due to the continued increase of the degree of infected nodes. To determine the outbreak spreading rates, we consider the equations for $\dot{I}$ and $\dot{[SI]}$ shown in (15). When $I \ll 1$, the non-negligible terms are

$$\begin{aligned} \dot{I} &= \beta[SI] \\ \dot{[SI]} &= \beta[SI]\,(k_0 - 1) + I\Delta. \end{aligned} \tag{16}$$

This coupled system of ordinary differential equations can be rewritten using the compound variable $\mathbf{y} = (I, [SI])^T$, giving the simple equation $\dot{\mathbf{y}} = \mathbf{A}\mathbf{y}$, where

$$\mathbf{A} = \begin{pmatrix} 0 & \beta \\ \Delta & \beta(k_0 - 1) \end{pmatrix} \tag{17}$$

The eigenvalues of $\mathbf{A}$ are $\lambda_\pm = \frac{1}{2}\left[\beta(k_0-1) \pm (4\Delta\beta + \beta^2(k_0-1)^2)^{1/2}\right]$. At long times, both I and $[SI]$ grow exponentially as $\exp(\lambda_+ t)$, and the time scale for this behavior to take hold is $1/(\lambda_+ - \lambda_-)$.

Even though infected individuals keep acquiring new links and their degree grows without bound, the rate of growth of the epidemics saturates at λ_+ because the newly-infected individuals start with the background number of neighbors, k_0. Therefore, the degree of the typical infected individual will grow to a steady state value in the exponential growth phase of the epidemic.

Fixation dynamics To analyze fixation dynamics we focus on the following equations in which we assume that $[SS]/S$ is negligible:

$$\begin{aligned} \dot{S} &= -\beta[SI] \\ \dot{[SI]} &= -\beta[SI]\left(\frac{[SI]}{S}+1\right) + \Delta\frac{S}{S+I\alpha}. \end{aligned} \tag{18}$$

If targeting is imperfect ($\alpha > 0$), when S becomes small enough that $S < \alpha$, the last term in (18) will be approximately equal to $\Delta S/\alpha$. Using this substitution, we get the following equation for the time evolution of the variable $x = [SI]/S$:

$$\dot{x} = -\beta x + \frac{\Delta}{\alpha}. \tag{19}$$

This will eventually saturate to the steady state value $x^* = \Delta/(\alpha\beta)$, and the susceptible rate, governed by the equation $\dot{S} = -\beta x S$, will decay exponentially as $\exp(-\beta x^* t) = \exp(-\Delta \cdot t/\alpha)$. In contrast to the instantaneous link-addition model, the rate does not decay exponentially. Thus, the fraction of S decreases faster and as $t \to \infty$, $S \to 0$.

However, if $\alpha = 0$ (perfect targeting) then the last term of (18) is simply Δ. Unlike all other cases, the rate of susceptible individuals will become zero at a finite time. To see why this behavior is the solution to the differential equations when S approaches zero, we use the following *ansatz*:

$$\begin{aligned} S(t) &= S_0(t^*-t)^a \\ [SI](t) &= [SI]_0(t^*-t)^b. \end{aligned} \tag{20}$$

The first equation of (18) gives

$$-aS_0(t^*-t)^{a-1} = -\beta[SI]_0(t^*-t)^b, \tag{21}$$

implying that $a = b+1$. This also implies that near t^*, $[SI]/S \gg 1$, and therefore, the second equation of (18) gives

$$-b[SI]_0(t^*-t)^{b-1} = -\beta\frac{[SI]_0^2}{S_0}(t^*-t)^{b-1} + \Delta. \tag{22}$$

For both right-hand-side terms to be comparable, we need $b = 1$. Finally, we recover the prefactors $S_0 = \frac{1}{2}\beta\Delta$ and $[SI]_0 = \Delta$ from (21) and (22).

Interestingly, if α is small but nonzero, then as in the instantaneous link-adding scenario, the dynamics of the infection will cross over from a regime that behaves as if $\alpha = 0$, that is where S approaches zero quickly and appears headed to vanish at some finite time, to, once S becomes comparable to α, a regime of regular exponential decay with rate constant Δ/α.

Another interesting consequence is the difference in the continuous case between perfect targeting and even slightly flawed targeting ($\alpha > 0$). If the targeting is perfect, then all of the individuals will be infected in finite time for any positive Δ, the number of links created per unit time. In constrast, no matter how large the value of Δ, if α is non-zero, it still takes an infinite amount of time to infect all individuals. Error-prone infected individuals cannot convert the whole population in finite time as eventually the false-positive identifications dominate the true-positive ones as the fraction of susceptible individuals becomes increasingly small. Alternately, converting the entire population in finite time requires each infected individual to create a non-zero number of new links with susceptible individuals on average per time step. Of course, the time for the number of susceptible individuals to reach a small fraction of the population ($S < \epsilon$) will depend greatly on the link generation rate for either α regime.

3 Epidemics with utility benefits

General Dynamics In the main text, we showed how explicit preferences can lead to strategic assortative or disassortative rewiring. From this, we can derive the rates of change of I, S, $[SI]$, $[II]$ and $[SS]$. $[SI]$, $[II]$ and $[SS]$ are normalized to sum to the average number of edges per individual E/N.

Consider a population of size N with E edges. We assume a well-mixed population. The infection dynamics without rewiring are the same as in the connectivity benefits model, without recovery $r = 0$ or connectivity benefit $\Delta = 0$ (see Eq. 4 in the main text):

$$\begin{aligned}
\dot{I} &= \beta[SI] \\
\dot{[SI]} &= \beta[SI]\left(2\frac{[SS]}{S} - \frac{[SI]}{S} - 1\right) \\
\dot{[II]} &= \beta[SI]\left(\frac{[SI]}{S} + 1\right)
\end{aligned}$$

and $S = 1 - I$, $[SS] = \frac{E}{N} - [SI] - [II]$.

Infected individuals that rewire disassortatively do so at a rate:

$$r_{i-i\to s} = \frac{I}{E}(1-(1-\alpha_{II})^{k_I})$$

This is the proportion of infected individuals that have at least one II link they wish to replace by an SI link. Here $\alpha_{II} = \frac{2[II]}{[SI]+2[II]}$ is the probability that a stub coming out of an infected node is an II stub, and $k_I = E\frac{[SI]+2[II]}{NI}$ is the average degree of infected nodes.

Infected individuals that rewire assortatively do so at a rate:

$$r_{i-s\to i} = \frac{I}{E}(1-(1-\alpha^I_{SI})^{k_I})$$

$\alpha^I_{SI} = \frac{[SI]}{[SI]+2[II]}$ is the probability that a stub coming out of an infected node is an SI stub.

As we saw, for susceptibles, assortative and disassortative rewiring rates can depend on the number of infected neighbors. We use Eq. 6 and compute the probability that a susceptible node meets the condition for disassortative rewiring assuming $S_n = k_S - I_n$ where $k_S \equiv E\frac{[SI]+2[SS]}{NS}$ is the average degree of susceptible nodes. Susceptibles prefer assortative rewiring as long as $I_n < \hat{I}_n$, where

$$\hat{I}_n = \frac{-d_S/\beta - c_I + (d_I - d_S) - (b_I - b_S)k_S - (c_I - c_S)NI}{2(d_I - d_S)}$$

Therefore, the rewiring rates for susceptibles:

$$r_{s-i\to s} = \frac{S}{E}P(1 \le I_n \le \hat{I}_n) = \frac{S}{E}\sum_{j=1}^{\hat{I}_n}\binom{k_S}{j}(\alpha^S_{SI})^j(1-\alpha^S_{SI})^{k_S-j}$$

$$r_{s-s\to i} = \frac{S}{E}P(1 \le S_n \le k_S - \hat{I}_n) = \frac{S}{E}\sum_{j=1}^{k_S-\hat{I}_n}\binom{k_S}{j}(\alpha_{SS})^j(1-\alpha_{SS})^{k_S-j}$$

where $\alpha_{SS} = \frac{2[SS]}{[SI]+2[SS]}$ and $\alpha^S_{SI} = \frac{[SI]}{[SI]+2[SS]}$.

We use these rates and the analysis of predicted utility in the main text to derive dynamics corresponding to the three cases considers: *evangelizers*, *cool kids*, and *snobs*.

Evangelizers case The dynamics in the evangelizers case is given by the following ODEs:

$$\dot{I} = \beta[SI]$$

$$\dot{[SI]} = \beta[SI]\left(2\frac{[SS]}{S} - \frac{[SI]}{S} - 1\right) + \frac{I}{E}(1-(1-\alpha^I_{II})^{k_I}) + \frac{S}{E}(1-(1-\alpha_{SS})^{k_S})$$

$$\dot{[II]} = \beta[SI]\left(\frac{[SI]}{S} + 1\right) - \frac{I}{E}(1-(1-\alpha^I_{SI})^{k_I})$$

To examine the breakout dynamics, consider the initial situation where $I \ll 1$, $[SI]^2 \approx 0$, $[SS]/S \approx 1$, $\alpha_{SS} \approx 1$, $\alpha^I_{II} \approx 0$. We can simplify this system to:

$$\dot{I} = \beta[SI]$$
$$\dot{[SI]} = \beta[SI] + 1/E$$

Initial growth is thus exponential.

To examine the fixation dynamics, consider the limiting situation where $S \ll 1$, $[SS] \approx 0$, $\alpha_{SS} \approx 0$, $\alpha^I_{II} \approx 0$. We can now simplify this system to:

$$\dot{I} = \beta[SI]$$
$$\dot{[SI]} = -\beta[SI]\left(\frac{[SI]}{S} + 1\right) + I/E$$

These equations take the same form as Eq. (18) when $\alpha = 0$ (continuous link addition with perfect targeting), which was shown to correspond to super-exponential fixation.

Cool kids case The cool kids case gives the following ODEs:

$$\dot{I} = \beta[SI]$$
$$\dot{[SI]} = \beta[SI]\left(2\frac{[SS]}{S} - \frac{[SI]}{S} - 1\right) - \frac{I}{E}(1-(1-\alpha^I_{SI})^{k_I}) + \frac{S}{E}(1-(1-\alpha_{SS})^{k_S})$$
$$\dot{[II]} = \beta[SI]\left(\frac{[SI]}{S} + 1\right) + \frac{I}{E}(1-(1-\alpha^I_{SI})^{k_I})$$

Snobs case In the snobs case $\hat{I}_n = \frac{S_n+2}{3} = \frac{k_S+2}{4}$. The dynamics are described by the following equations:

$$\dot{I} = \beta[SI]$$
$$\dot{[SI]} = \beta[SI]\left(2\frac{[SS]}{S} - \frac{[SI]}{S} - 1\right) - \frac{I}{E}(1-(1-\alpha^I_{SI})^{k_I})$$
$$- \frac{S}{E}\sum_{j=1}^{(k_S+2)/4}\binom{k_S}{j}(\alpha^S_{SI})^j(1-\alpha^S_{SI})^{k_S-j} + \frac{S}{E}\sum_{j=1}^{k_S-(k_S+2)/4}\binom{k_S}{j}(\alpha_{SS})^j(1-\alpha_{SS})^{k_S-j}$$
$$\dot{[II]} = \beta[SI]\left(\frac{[SI]}{S} + 1\right) + \frac{I}{E}(1-(1-\alpha^I_{SI})^{k_I})$$

Agent-based version: robustness of mean-field results to stochasticity and heterogeneity The ODE model can be seen as a mean-field approximation of a discrete stochastic epidemic-spreading

process, one with local differences in connectivity, degree heterogeneity, and correlated network properties. In order to check the robustness of the mean-field approximation, here we present simulation results of a stochastic discrete agent-based version of the same basic model.

In the agent-based model, agents have the same utility function as described in the main text. N agents are distributed on a network with E edges, which starts off as Erdős-Rényi random graph. At each time step, a randomly selected individual first attempts to adaptively rewire according to its state and neighborhood (following equations Eq. 5 and Eq. 6 of the main text). Afterward, each infected-susceptible edge leads to the spread of an infection with probability β. Figure S7 compared the results of the ODE model with the results of the agent-based simulations, for each of the three cases defined in the main text as well as a no-rewiring condition. Parameters are set same as in the main text (population of $N = 1000$ agents, $E = 2000$ edges, initial infection rate 0.05, $\beta = 5 \times 10^{-4}$). We see that the dynamics are qualitatively the same in both the discrete agent-based version and the continuous mean-field version.

The largest quantitative difference between the agent-based and mean-field dynamics is seen for the snobs case. For both kinds of dynamics, the final population is characterized by two disconnected components, where susceptible individuals are only connected to other susceptible and infected individuals are only connected to other infected. However, the final portion of infected is somewhat larger for agent-based dynamics (≈ 0.7) than for mean-field dynamics(≈ 0.6). This difference arises because the agent-based dynamics witness an initial "bump" in the number of susceptible-infected connections (blue line, right subplot of bottom row of Fig. S7), which is much less pronounced under the mean-field dynamics (blue line, right subplot of middle row of Fig. S7). This initial growth in susceptible-infected connections provides an early boost to the rate of infected spread under agent-based dynamics (black line, right subplot of top row of Fig. S7).

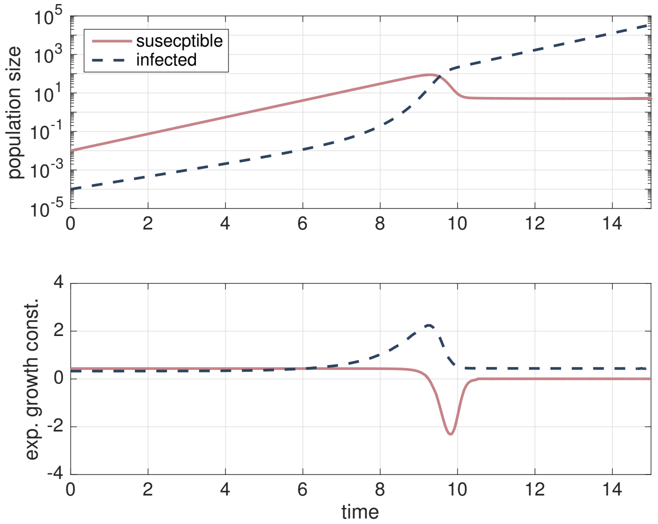

Figure S1: **Sample dynamics of model 1 with imperfect transmission.** Numerical solution of Eq. 1 with parameters $\beta = .05$, $s = .01$, and $p = .75$.

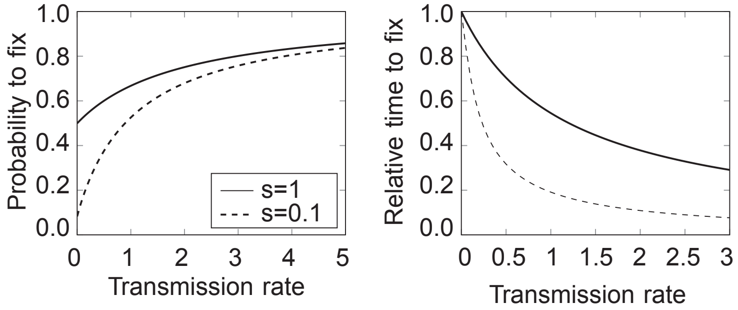

Figure S2: **Probability of fixation in the evolutionary model.** The probability that a novel bene fixes (completely saturates the population) as a function of the infection rate, β, when $s = 1$ and $s = 0.1$ (left panel). The time to reach a stationary state (where the bene either fixes or goes extinct) as a function of β, when $s = 1$ and $s = 0.1$ (right panel). Time is plotted relative to the time it takes a match model where $\beta = 0$. $N = 100$ in all cases.

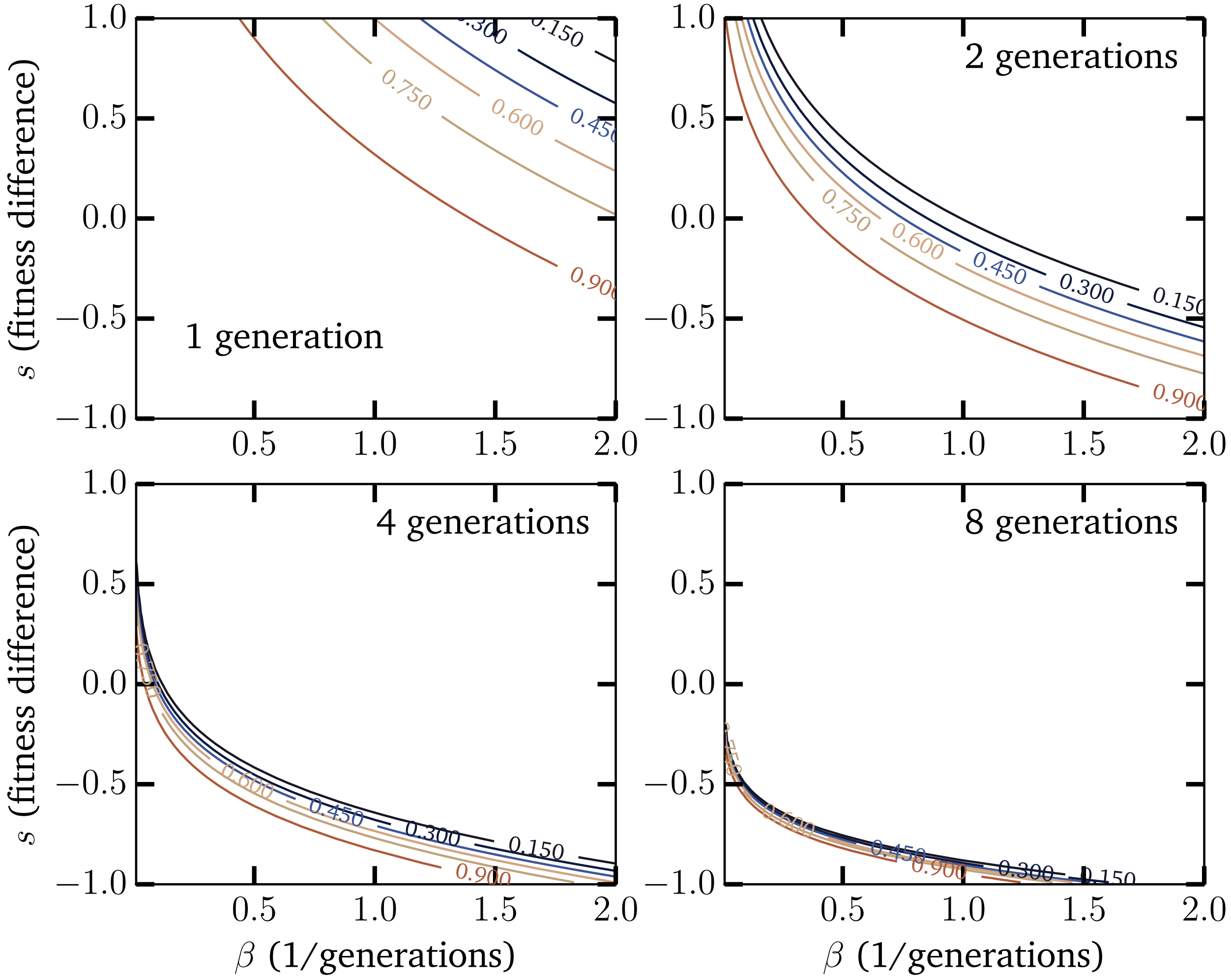

Figure S3: **Dynamics of our closed form solution for epidemics with fitness benefits.** Our analytical solution assumes a perfect vertical transmission (i.e. $p = 1$) and neglects the interplay of horizontal and vertical transmissions. We then explore the role of the horizontal transmission rate β and of the fitness impact of the bene s by showing contours of the fraction of uninfected individuals in the population at different time. Since time is defined as the number of generations, we show: (top left) time $t = 1$, (top right) time $t = 2$, (bottom left) time $t = 4$ and (bottom right) time $t = 8$.

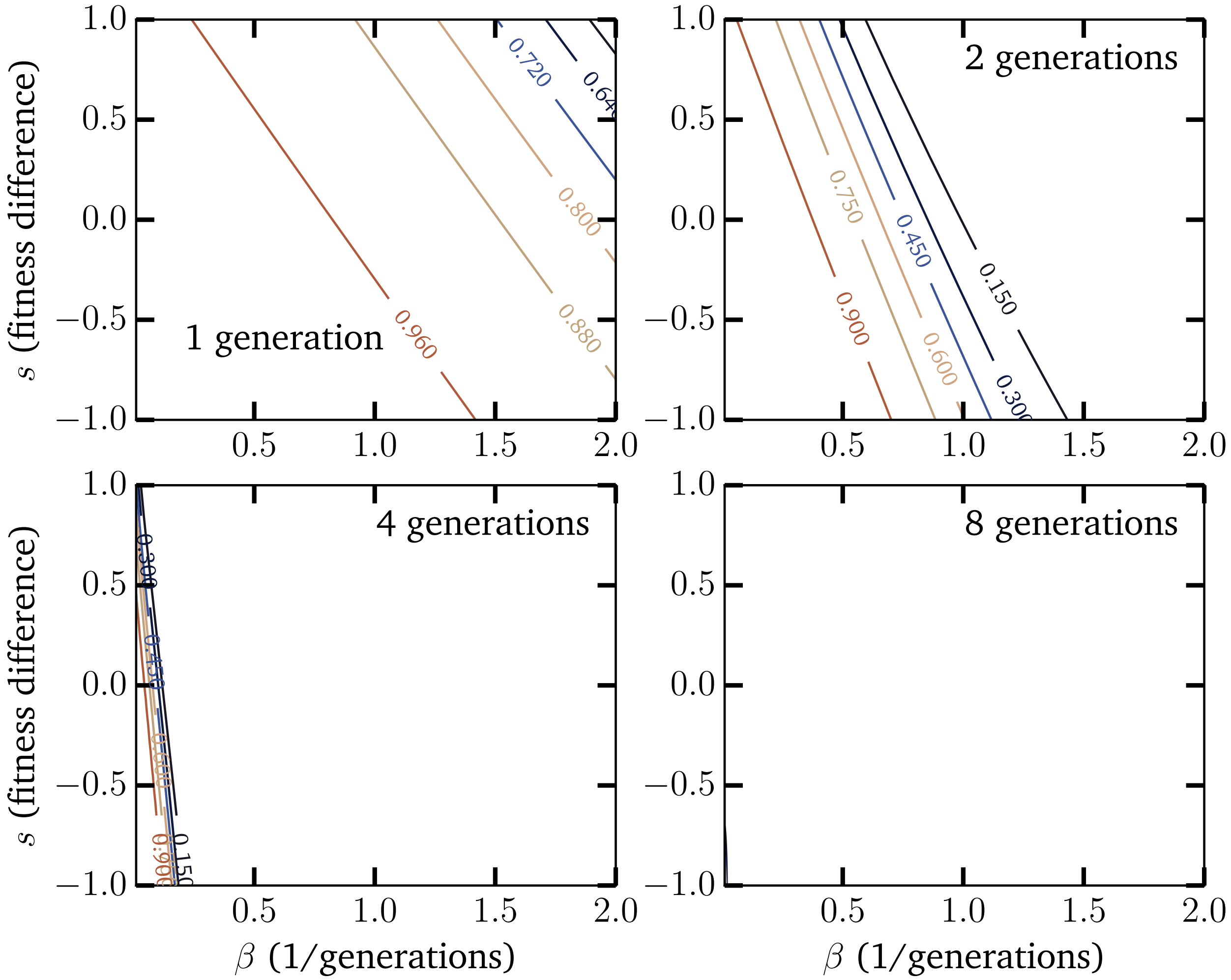

Figure S4: **Dynamics of epidemics with fitness benefits with perfect vertical transmission.** We compare the results of our analytical solution shown in Fig. S3 to numerical integration of the system of ordinary differential equations. Using a perfect vertical transmission $p = 1$, this numerical solution now allows us to observe the interplay of horizontal and vertical transmissions. We first see that the analytical solution only approximates the system correctly at high fitness benefit, i.e. s close to 1. In fact, it performs terribly at low bene fitness, $s \to -1$, where it systematically *underestimates* the speed at which the bene invades the population.

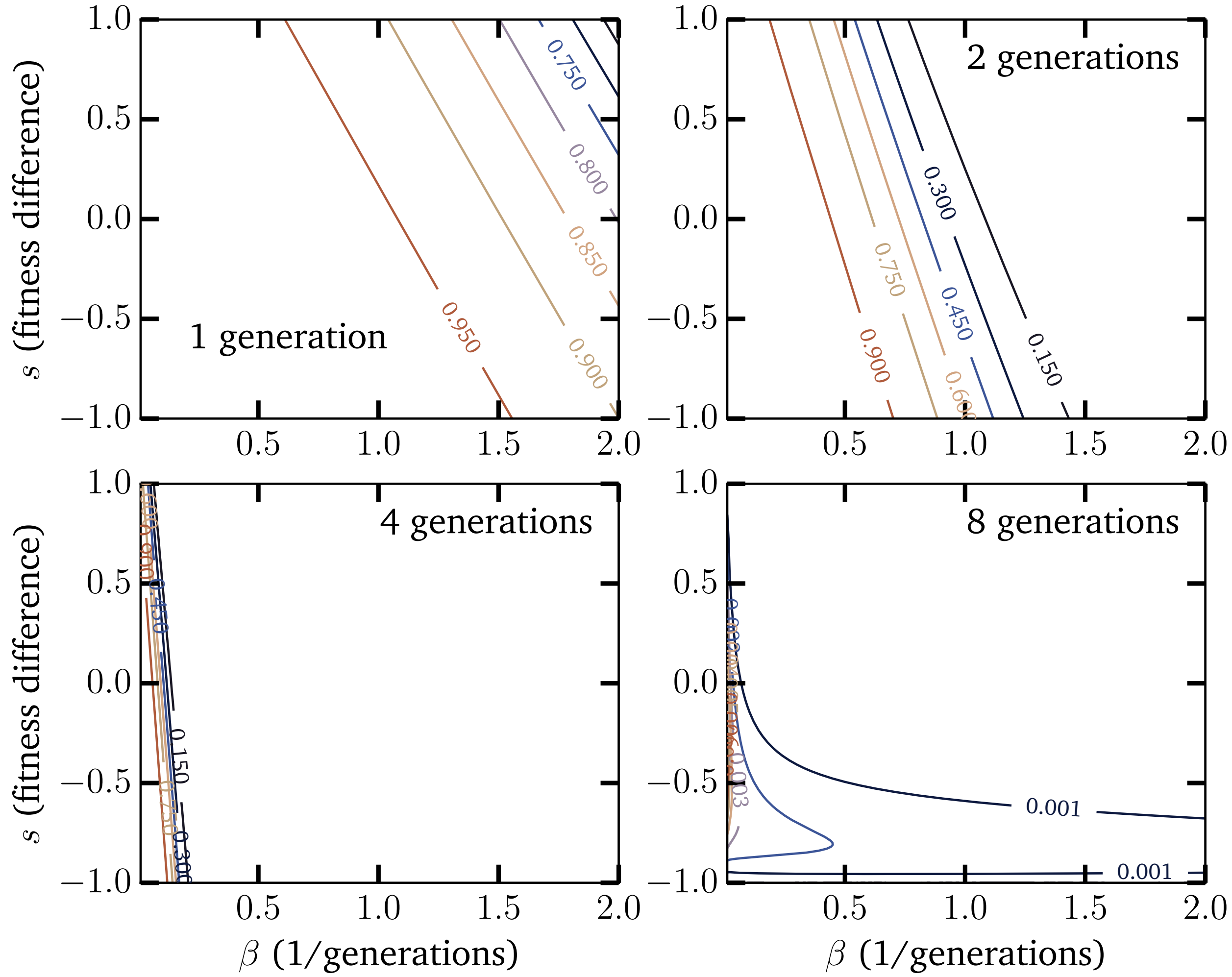

Figure S5: **Dynamics of epidemics with fitness benefits with imperfect vertical transmission.** We reproduce the numerical analysis used in Fig. S4 but now with imperfect vertical transmission: $p = 0.8$. As expected, we find a generally slower invasion of the bene in the population since the infected individuals now replenish the pool of uninfected individuals. Interestingly, we also find non-monotonous behaviour in parameter space. In essence, there is a tradeoff between a decrease in s slowing down vertical transmission of the bene but also replenishing susceptible individuals to be infected horizontally.

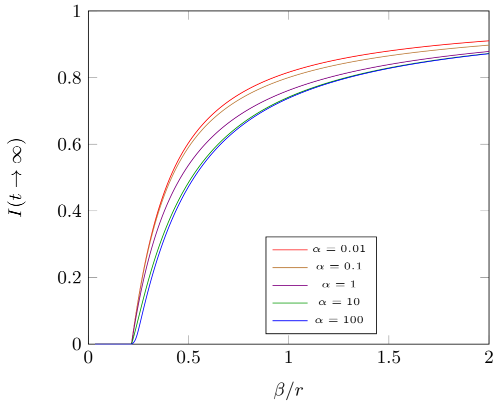

Figure S6: **Steady state size of the infected population in the case of instantaneous link addition.** The figure shows the value of the equilibrium value of I as a function of β/r when $k_0 = 3$ and $\Delta = 2$. When $\beta < \beta_c$ (here $\beta_c = r/5$), the only possible equilibrium is $I = 0$. When $\beta > \beta_c$, the equilibrium state $I = 0$ becomes unstable, and a steady equilibrium with $I > 0$ emerges.

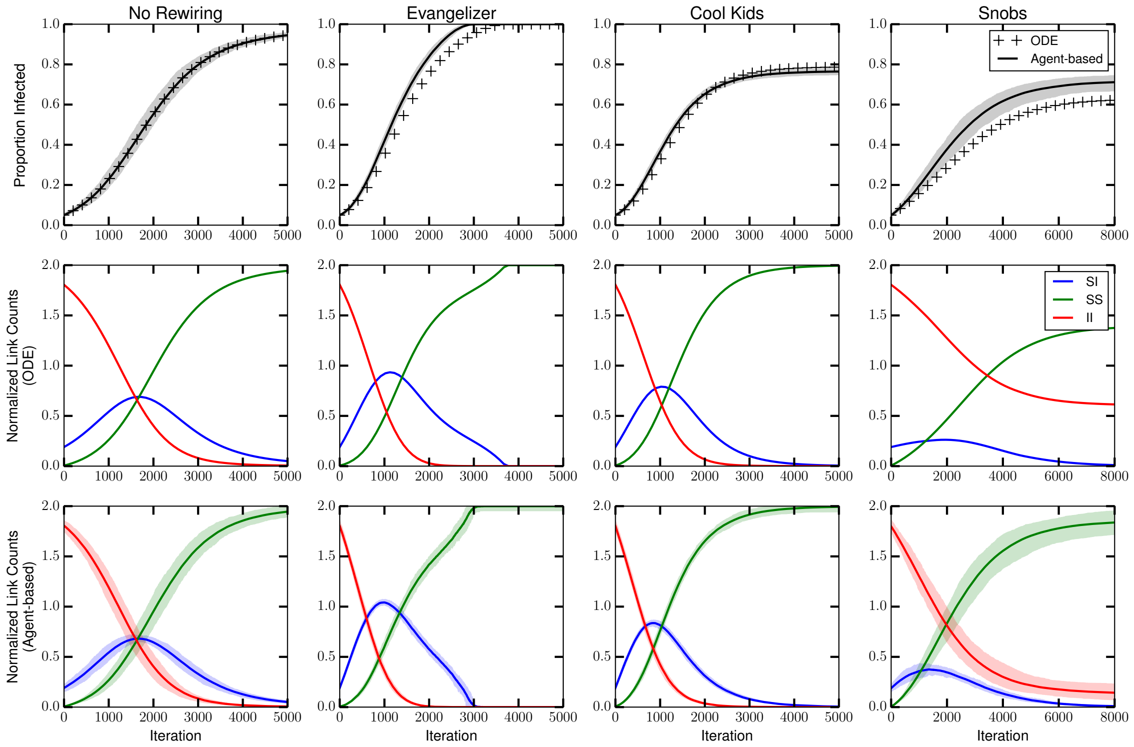

Figure S7: Comparison between the agent-based model and the mean-field approximation (ODE model) presented in the main text. For both models, parameters are $N = 1000$, $E = 2000$, initial infection rate 0.05, $\beta = 5 \times 10^{-4}$. Top row: the proportion of infected individuals in the agent-based model (black line) over the analogous ODE result (crosses). Middle row: the portion of links corresponding to susceptible-infected (blue), susceptible-susceptible (green), and infected-infected (red) connections in the ODE model. Bottom row: the portion of links corresponding to susceptible-infected (blue), susceptible-susceptible (green), and infected-infected (red) connections in the agent-based model. For the agent-based results in the top and bottom rows, the solid line indicates the mean, and the shaded area represents the 10% to 90% percentiles across 100 runs. Link counts (middle and bottom row) are normalized to sum to the average number of edges per individual, E/N.
